# Primary MALT Type Skin Lymphoma—Is ‘Wait and See’ a Possible Strategy?

**Published:** 2008-03-19

**Authors:** Florentina Silvia Delli, Thomas Zaraboukas, Ioanna Mandekou-Lefaki

**Affiliations:** 1 Dermatology Department, Private Practice, Thessaloniki, Greece; 2 Anatomopathology Department, Aristotle University of Thessaloniki, Greece; 3 Dermatology Department, State Hospital for Skin and Venereal Diseases, Thessaloniki, Greece

**Keywords:** primary cutaneous B-cell lymphoma, MALT type, therapy

## Abstract

Primary cutaneous lymphomas are the second most common site of extranodal non-Hodgkin lymphoma. A specifically type named extranodal marginal zone B-cell lymphomas are indolent low-grade neoplasma.

We report a case of a 42-year-old white man with multiple subcutaneous tumors located on the trunk and neck. The histopathological exam showed a non-epidermotropic, dense lymphocytic infiltrate. Histologic, immunohistochemical and cytologenetic analysis diagnosed primary cutaneous B-cell lymphoma MALT type. Investigation for other extranodal MALT lymphoma gastrointestinal tract, lung, salivary and thyroid glands was negative. The patient refused radiotherapy, but he accepted every 6 months close follow-up. Over a seven years period, we noticed a progressively disappearance of the skin lesions.

The necessity of aggressive treatment of this disease with excellent prognosis is discussed.

The treatment necessity of primary cutaneous B-cell lymphoma MALT type is discussed.

## Introduction

Extranodal marginal zone B-cell lymphomas [EMZL, mucosa-asociated lymphoid tissue (MALT) lymphomas] are typically indolent low-grade neoplasma that can develop in a variety of sites from reactive infiltrates associated with chronic infections or autoimmune processes.

Primary cutaneous B-cell lymphomas (PCBCLs) comprise approximately 20% of cutaneous lymphomas (Zackheim et al. 2000). The low-grade subtype is separated in marginal zone (MZL) and follicle center lymphomas according to the recent World Health Organization—European Organization for Research and Treatment of Cancer classification, with distinct histologic and immuohistochemical profiles (Burg et al. 2005).

In a recent study (de la Fourchardin et al. 2005) MZL showed to have a good prognosis, although transformation can occur, needing more aggressive treatment.

We report a case that cured without any therapeutical intervention.

## Case Report

**Figure 1:**
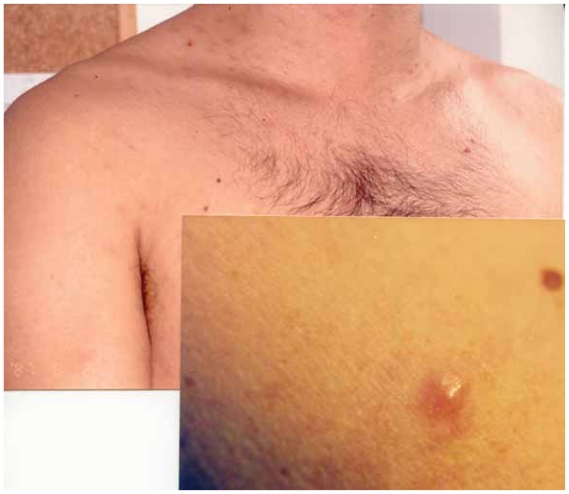
Multiple skin lesions on the neck, trunk and upper arms, consisting of infiltrated deep red violaceus nodules measuring 1–1,5 cm.

**Figure 2:**
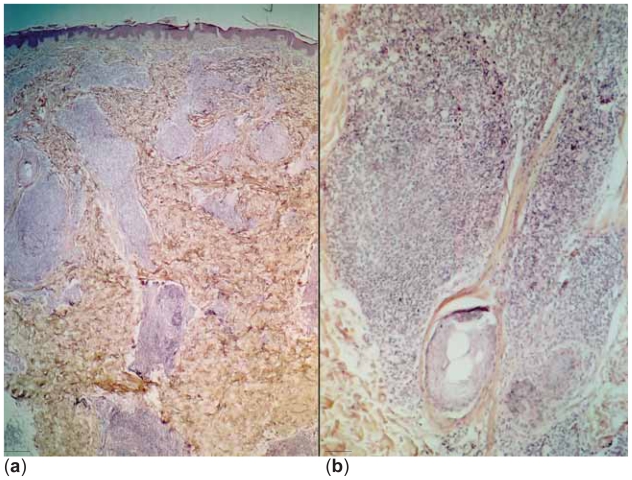
(**a**) Diffuse infiltrate sparing the epidermis mainly composed of small lymphocytes. (**b**) The same Figure—higher magnification.

**Figure 3:**
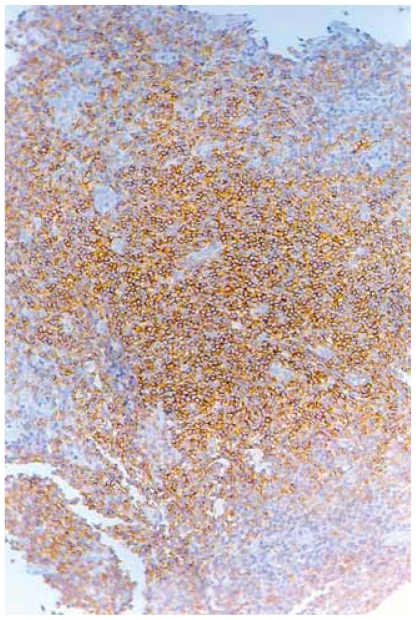
Small CD20+ B cells.

**Figure 4:**
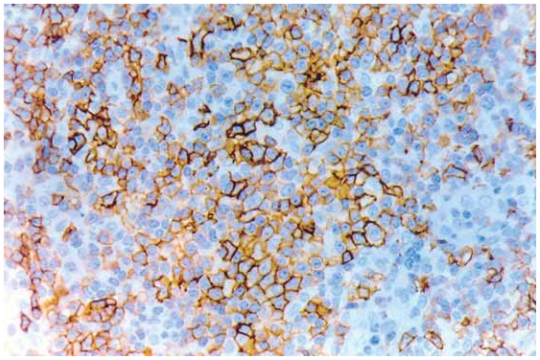
CD10 antigen stain.

A 42-year-old man presented with multiple skin lesions on the neck, trunk and upper arms, consisting in infiltrated deep red violaceus nodules measuring 1–1,5 cm (Fig. 1). The lesions had appeared 8 months before. The patient is a hepatitis C carrier, without any biochemical sign of chronic active hepatitis. Histopathological examination showed a diffuse infiltrate sparing the epidermis (Fig. 2). The central area infiltrate contains mainly small CD20+ (Fig. 3) and CD45RA+ B cells. We used molecular analysis for detecting if there existed clonal proliferation of lymphoid cells and it had showed to be negative. Staining for Bcl-6, CD10 (Fig. 4), CD21 and CD45RA were positive. A small number of follicular and extrafollicular B cells showed expression of Bcl-2 protein. The t(14; 18) translocation with RTQ-PCR (Taqman) method was not found. Polymerase chain reaction (PCR) for Borrelia burgdorfer flagellin gene was negative in formalin-fixed paraffin-embedded tisuue of skin biopsy. Investigation for other nodal and extranodal MALT lymphoma was negative. Laboratory tests, including Borrelia burgdorferi serology and Helicobacter pylori, were all normal, apart from confirming the previously diagnosed hepatitis C. Other investigations including CT scan of the chest, abdomen and pelvis, showed no abnormalities. The bone marrow exam was negative. These findings confirmed the clinical and histopathological diagnosis of primary marginal zone cutaneous B-cell lymphoma.

In order to perform further control of the patient liver’s function, he was advised to consult a gastrenterologist. Liver function and the general check-up of all gastrenterologist system were founded at physiological state.

**Figure 5:**
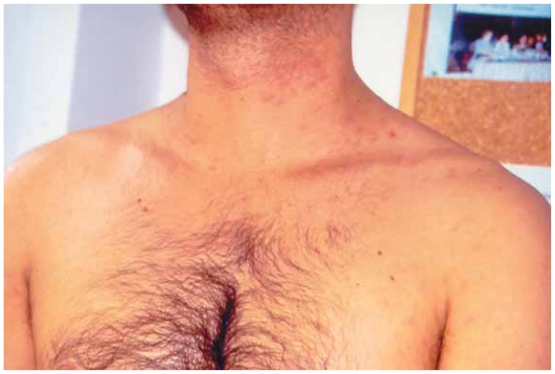
The skin lesions disappeared leaving a slight hyperpigmentation.

Because of the persistence of the skin lesions, we proposed radiotherapy. The patient refused it. He agreed every 6 months close clinical follow-up. Over a seven years period, we noticed a progressively disappearance of the skin lesions, leaving only a slight hyperpigmantation (Fig. 5).

## Discussion

Classification of lymphomas has always been a challenge because the nomenclature has changed many times over the past several decades to reflect changes in our knowledge about the immunology and pathogenesis of these neoplasms. Although criteria for confirming the primary nature of a cutaneous lymphoma have varied, a current working definition would be: a lymphoma which is determined to be confined to the skin after physical examination, chest/abdomen/pelvis imaging studies, blood smear review, and bone marrow staging biopsies.

A major advance in our understanding of cutaneous B-cell lymphomas came from studies that began to separate primary from secondary forms. Since most series tended to group all cutaneous lymphomas and since PCBCL is a rare disorder, this seemingly basic difference tended to be overlooked. Perhaps no other advance in the characterization of extranodal low-grade B-cell lymphomas has been as significant in the past 20 years as the concept of extranodal marginal-zone B-cell MALT lymphomas, first introduced in 1983 (Isaacson and Wright, 1983; Isaacson 1999).

Burke et al. were among the first to describe entities that are now considered characteristic of PCBCL (Burke et al. 1981; Santucci et al. 1991; Yang et al. 2000). The extension of MALT type lymphomas concept to the skin in the form of skin-associated lymphoid tissue and so-called immunocytomas of the skin helped define a new type of PCBCL. Recognition of this type of lymphoma set the stage for modern classifications and definitions of PCBCL.

The typical immunophenotype is CD20+, CD5, CD10 with immunoglobulin (Ig) light chain restriction. Monoclonal Ig gene rearrangement can be demonstrated by molecular genetic methods such as Southern blot or PCR. Recent studies conclude that known recurrent chromosomal abnormalities rarely occur in primary cutaneous MZLs and suggest the possibility of a variety of initial oncogenic events leading to a common downstream pathway (de la Fouchardiere et al. 2006).

EMZL are thought to develop from reactive infiltrates that represent immune responses to external or autoantigens. Except for gastric EMZL, the antigenic triggers of EMZL development are mostly unknown, although a subset of cutaneous EMZL have been associated with Borrelia burgdorferi infections (Slader, 2001) and, recently with gastric Helicobacter pylori infection (Lefaki et al. 2006). In our case none of these pathological agents was detected.

Like their counterparts in other extranodal sites, the behavior of EMZL is indolent (Cerroni et al. 1997; de la Fouchardiere et al. 2005). The recent WHO/EORTC classification (Burg et al. 2005) reviews the histological, pheotypical, and molecular genetic features and reminds that these findings always have to be interpreted in the context of the clinical features and biological behaviour.

First-line therapy includes excision, local irradiation and chemotherapy. Recently were successfully tried intralesional anti-CD20monoclonal antibody rituximab (Kyrtsonis et al. 2006) and IFNalpha2a (Cozzio et al. 2006).

The most PCBCL MALT type has an excellent prognosis. Cutaneous recurrence is common, but systemic dissemination and death from disease are uncommon. For patients with PCMZL who have multifocal lesions, chlorambucil therapy and radiotherapy are suitable therapeutic options. In case of cutaneous relapses, the beneficial effects of treatment should carefully be weighed against the potential adverse effects (Hoefnagel et al. 2005).

We report a case of a young male with multiple lesions. The diagnosis was made based on WHO classification. Without any therapeutical intervention, the lesions disappeared over a seven years period. The known excellent prognosis and the indolent course of the disease in our patient, in association with close clinical follow-up succeed the cure in our case.

## Conclusion

MZL, the predominant type of PCBCL, is having a good prognosis, although transformation can occur, needing a more aggressive treatment. The clinical evolution of our case suggests that in selected cases of histopathological and immunological proved MALT-type PCBCL, the “wait and see” strategy might be eligible, always with very close follow-up of the patient.
